# Supplementary material for: Risk and prognostic nomograms for hepatocellular carcinoma with newly-diagnosed pulmonary metastasis using SEER data
Source: PeerJ. 2019 Aug 16;7:e7496. doi: 10.7717/peerj.7496 (PMC6699481; doi:10.7717/peerj.7496)
Supplement: Supplemental Information 1 [file peerj-07-7496-s001.docx]

| **Inclusion criteria and exclusion criteria** | **Program selection codes** |
| --- | --- |
| Included cases of liver cancer diagnosed from 2010 to 2015 | Included {Year of diagnosis} = ‘2010’, ‘2011’, ‘2012’, ‘2013’, ‘2014’, ‘2015’ AND {Site recode ICD-O-3/WHO 2008} = ‘Liver’ |
| Included patients with hepatocellular carcinoma | Included {Histologic Type ICD-O-3} = ‘8170’, ‘8171’, ‘8172’, ‘8173’, ‘8174’, ‘8175’ |
| Included liver cancer as the first primary malignant tumor | Included {Sequence number} = ‘one primary only’ or ‘1st of 2 or more primaries’ |
| Excluded patients with unknown follow-up time | Excluded {Survival months} = ‘Unknown’ |
| Excluded patients with unknown cause of death | Excluded {SEER cause-specific death classification} = ‘Dead (missing/unknown COD)’ |
| Excluded patients with unknown race | Excluded {Race recode (White, Black, Other)} = ‘Unknown’ |
| Excluded patients with unknown T stage | Excluded {Derived AJCC T, 7th ed (2010+)} = ‘NA’ |
| Excluded patients with unknown surgical approach | Excluded {RX Summ--Surg Prim Site (1998+)} = ‘90’, ‘99’ |
| Excluded patients with unknown brain metastasis | Excluded {CS mets at DX-brain (2010+)} = ‘N/A’, ‘Unknown’ |
| Excluded patients with unknown bone metastasis | Excluded {CS mets at DX-bone (2010+)} = ‘N/A’, ‘Unknown’ |
| Excluded patients with unknown intrahepatic metastasis | Excluded {CS mets at DX-liver (2010+)} = ‘N/A’, ‘Unknown’ |
| Excluded patients with unknown pulmonary metastasis | Excluded {CS mets at DX-lung (2010+)} = ‘N/A’, ‘Unknown’ |
