# Supplementary figures and images for: Risk and prognostic nomograms for hepatocellular carcinoma with newly-diagnosed pulmonary metastasis using SEER data
Source: PeerJ. 2019 Aug 16;7:e7496. doi: 10.7717/peerj.7496 (PMC6699481; doi:10.7717/peerj.7496)

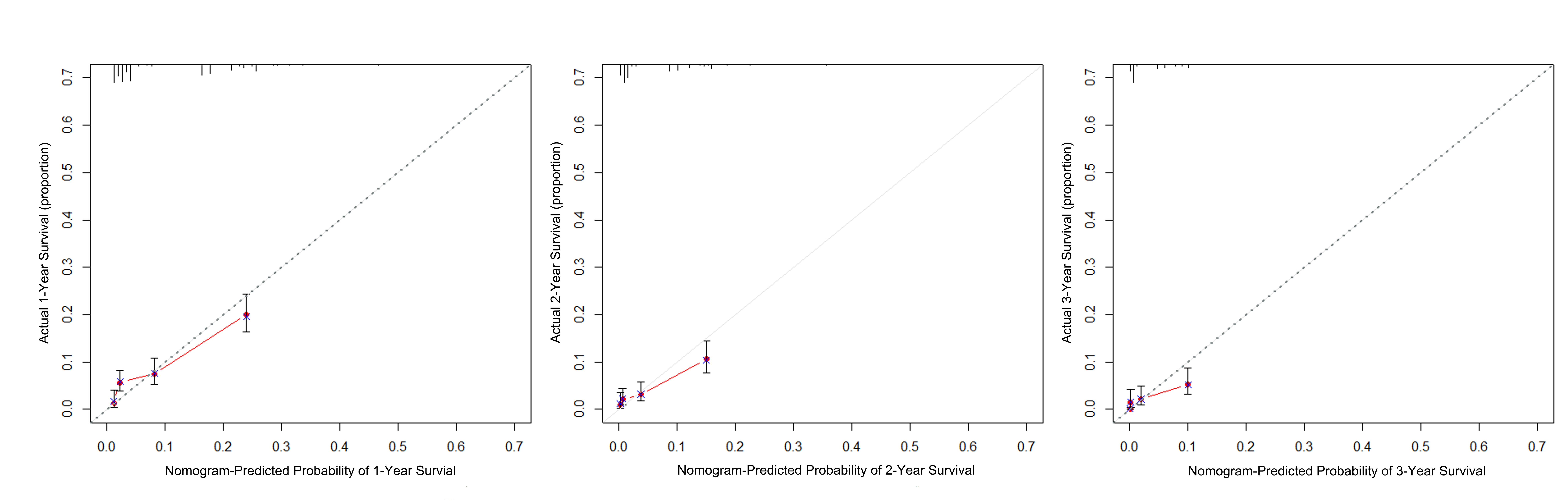

Supplement: Supplemental Information 4 — (A) Calibration curves of the nomogram prediction of 1-year overall survival of patients with pulmonary metastasis from hepatocellular carcinoma. (B) Calibration curves of the nomogram prediction of 2-year overall survival of patients with pulmonary metastasis from hepatocellular carcinoma. (C) Calibration curves of the nomogram prediction of 3-year overall survival of patients with pulmonary metastasis from hepatocellular carcinoma. [file peerj-07-7496-s004.png]
